# First-principles study of electronic structures and stability of body-centered cubic Ti–Mo alloys by special quasirandom structures

**DOI:** 10.1088/1468-6996/15/3/035014

**Published:** 2014-06-27

**Authors:** Ryoji Sahara, Satoshi Emura, Seiichiro Ii, Shigenori Ueda, Koichi Tsuchiya

**Affiliations:** 1 National Institute for Materials Science (NIMS) 1-2-1 Sengen, Tsukuba 305-0047, Japan; 2 Scynchrotron X-ray Station at SPring-8, National Institute for Materials Science (NIMS), 1-1-1 Kouto, Sayo-cho, Sayo-gun, Hyogo 679-5148, Japan

**Keywords:** first-principles calculation, *β* Ti–Mo alloy, solid solution system, special quasirandom structures, hard x-ray photoelectron spectroscopy

## Abstract

The electronic structures and structural properties of body-centered cubic Ti–Mo alloys were studied by first-principles calculations. The special quasirandom structures (SQS) model was adopted to emulate the solid solution state of the alloys. The valence band electronic structures of Ti–Mo and Ti–Mo–Fe alloys were measured by hard x-ray photoelectron spectroscopy.

The structural parameters and valence band photoelectron spectra were calculated using first-principles calculations. The results obtained with the SQS models showed better agreement with the experimental results than those obtained using the conventional ordered structure models. This indicates that the SQS model is effective for predicting the various properties of solid solution alloys by means of first-principles calculations.

## Introduction

Interest in and demand for *β* titanium alloys are growing due to their high specific strength [1], good formability, and excellent biocompatibility [2]. Since most *β*-Ti alloys contain large amounts of alloying elements to control the phase stability and microstructures [1, 2], it is important to clarify the role of each element to be able to design alloys which use alloying elements in the most efficient way. Ti–Mo alloys are prototypical *β* titanium alloys. It has been reported that Ti–15 mass % Mo alloy, which was developed as a corrosion-resistant titanium alloy during the 1950s [3], exhibited extremely high crevice corrosion resistance in simulated boiling sea water [4]. Also, the authors reported that the substitution of Fe for Mo improves the tensile strength of the Ti–15Mo alloy without compromising its high crevice corrosion resistance [5, 6].

Understanding the theories governing the structural, electronic, and elastic properties of compounds allows effective control of these properties. However, there have been few theoretical works in that direction. This is because *β* Ti–Mo alloys are all solid solution systems with a body-centered cubic (bcc) lattice in the high temperature region. One of the common problems in examining solid solution systems by first-principles calculations is the question of how to emulate random configurations within a limited number of atoms.

There are several approximations used in first-principles calculations for solid solution systems, for example, the virtual crystal approximation (VCA) method proposed by Bellaiche and Vanderbilt [7]. In VCA, a pseudopotential is generated as the weighted average of the pseudopotentials for each atomic species. VCA can describe compounds containing atoms of similar atomic size, in which there is only a small lattice distortion. However, it is not suitable for alloys containing atoms of vastly different sizes, where lattice relaxation effects may be significant. Using VCA, Chrzan *et al* studied the dislocation core structures for bcc Ti alloys [8]. They showed the difference in the dislocation core structures of 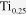 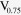 and 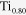 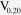 according to the ideal shear strength.

Ordered structures also have been used to analyze the properties of solid solution systems in first-principles calculations. Ikehata *et al* analyzed the electronic and elastic properties of Ti-based binary alloys with low elastic modulus [9]. They found that controlling the number of valence electrons is important for realizing Ti binary alloys with a low Youngʼs modulus.

In those studies, the randomness of atomic distribution, which might affect the electronic as well as the elastic properties, was not taken into account.

The Youngʼs modulus of *β* Ti–Mo alloys were theoretically estimated by introducing 16-atom systems, in which Ti atoms were replaced by Mo atoms to describe nonstoichiometric concentrations [11, 12]. These values were compared with the experimental values.

Recently the lattice parameters and relative stability of the 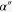 phase in Ti–*X* alloys with *X* = Ta, Nb, V, and Mo are investigated using the coherent potential approximation to deal with random alloys. Although it is shown that the properties were well analyzed, the local atomic distortion and the electronic properties depending on the atomic distribution cannot be treated, which is similar to VCA [13]. Although the phase stability of the 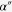 phase was studied relative to the *α* and *β* phases, the miscibility gap in the bcc phase was not described.

The special quasirandom structures (SQS) model proposed by Zunger *et al* [10, 14, 15] has also been used to analyze the properties of solid solution systems. In this model, randomness of atom distribution is introduced by emulating the correlation functions of an infinite random alloy within a finite supercell. The SQS model has been applied to many alloys, such as Cu–Pd systems [16], Ni–Pt and Cu–Au systems [17], Cu–Au, Ag–Au, Cu–Ag, and Ni–Au systems [18], Al–Cu–Mg–(Si) and Al–Zn–Mg systems [19], Mo–Nb, Ta–W, and Cr–Fe systems [20], seven hexagonal close-packed (hcp) binary alloys [21], 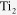 Al(C, N) systems [22], and eutectic Al–Ti alloys [23, 24]. Those studies discussed the performance of the SQS model for the description of structural, electronic, and elastic properties. However, there have been only a few analytical studies on bcc Ti alloys, such as Ti–Mo systems, where the different atom sizes may affect the electronic and structural properties.

In this work, we studied a series of bcc 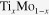 alloys with varying *x* to examine their electronic and structural properties through total energy calculations. In order to take into account the effects of random distribution of atoms, the SQS model was adopted. In section 2, we discuss our experimental and calculation procedures, and the results are presented and compared to experimental results in section 3. A summary of our work is given in section 4.

## Calculation and experimental procedures

### First-principles calculations using the SQS model

According to [10, 14, 15], in an SQS model, atomic correlation can be defined using the spin representation with occupation variable 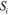 defined at each lattice site *i*. In the case of a bcc binary alloy 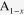 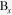, 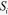 can take a value of −1 or +1 when *i* is occupied by atom A or B, respectively. Then, the correlation function is defined as 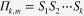 for a cluster of *k* sites spanning a distance *m*. The quantity 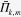 is obtained by averaging 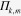 over all symmetry-equivalent clusters in the lattice. For a perfectly random solid solution, 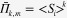 because occupation of a site is independent of occupation of other sites. For a bcc binary alloy, 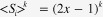. Therefore, 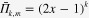. In the discussion below, this is represented as 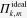.

SQS models with given number of atoms *N* were constructed by searching the configuration space and choosing the structure which minimizes 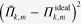 for all sets of *k* and *m*.

In the present study, SQS models were constructed using the Alloy Theoretic Automated Toolkit (ATAT) code [25, 26] using the following three sets of clusters *f* to compare the quality of atom distributions, where *k* is the number of atoms and *m* is the maximum distance: (1) 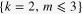, 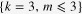, 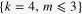, with 14 nonequivalent clusters, where *m* = 3 corresponds to 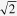 times the lattice constant; (2) 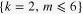, 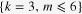, 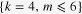, with 80 nonequivalent clusters, where *m* = 6 corresponds to two times the lattice constant; (3) 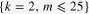, 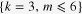, 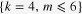, with 99 nonequivalent clusters and spanning the maximum distance in the direction of 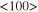 in the case of a 128-atom system. That is, *m* = 26 corresponds to four times the lattice constant, and therefore an atom is identical to its 26th nearest neighbor.

SQS models were generated for three nonstoichiometric concentrations: 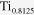 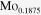, 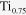 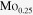, and 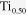 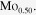 For comparison, ordered structure models for 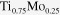 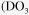 structure) and 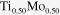 (B2 structure and B32 structure), as well as bcc Ti and bcc Mo systems (A2 structure), were also studied. It is a good starting point to demonstrate the SQS models by comparing with these structures (A2, 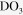, B2, and B32) because of the following reason: one of the conventional approaches to describe the ordering behavior for bcc binary alloys is a four-sublattice model with the second-nearest-neighbor interactions proposed by Allen and Cahn [27]. In the model, the four structures are enough to describe the tetrahedron clusters with the first and second-nearest-neighbor atoms. Since the tetrahedron cluster was high symmetry, a mixture of the tetrahedrons can also represent a disordered distribution of atoms on the bcc lattice.

Two systems of different sizes were constructed to examine the system size dependence of the calculation results. One was a 128-atom system constructed as a 4 × 4 × 4 simple-cubic supercell, and the other was a 384-atom system constructed as a 4 × 8 × 12 grid of primitive cells.

To perform density functional theory calculations, we used the projected augmented wave method [28, 29] as implemented in the Vienna *ab initio* simulation package [30, 31]. The exchange-correlation energy was calculated within the generalized gradient approximation (GGA) proposed by Perdew and Wang [32]. The total energy was minimized over the degrees of freedom of both the electron density and the ionic positions and structural parameters by the conjugate-gradient iterative minimization technique. Electronic convergence was set as 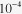 eV per atom for all the cases. The cut-off energy for the plane wave expansion was taken as 318.6 eV. Brillouin zone integrations were performed using a set of 4 × 4 × 4 *k*-points for the 128-atom systems and 4 × 2 × 2 *k*-points for the 384-atom systems. Tests were made using 6 × 6 × 6 and 8 × 8 × 8 *k*-points in the case of 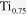 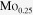 with the configuration in table 2(b) as an example, and confirmed that the final errors of the total energy difference were less than 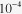 eV.

**Table 2.** Details of the SQS models for 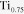 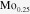 and 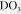 structure.

	System size	*f*	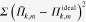	Formation energy, eV/atom
(II-a)	128	14	0.001 058	−0.040
(II-b)	128	80	0.075 116	−0.050
(II-c)	128	80	0.040 699	−0.042
(II-d)	128	80	0.018 385	−0.042
(II-e)	128	80	0.017 274	−0.040
(II-f)	128	99	0.022 488	−0.041
(II-g)	128	99	0.022 910	−0.040
(II-h)	384	80	0.007 450	−0.042
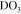 structure	128	80	27.250	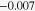

In order to analyze the phase stability of bcc Ti–Mo alloys, the formation energy was estimated as follows:

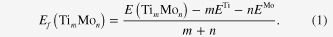
Here, 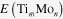 is the total energy of a 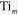 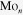 alloy with *m* Ti and *n* Mo atoms. 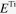 and 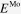 are the energies of Ti and Mo in their ground state phases, namely hcp Ti and bcc Mo.

The elastic properties were estimated as follows. The bulk modulus, *B*, was calculated using the Murnaghan equation of state [33]:

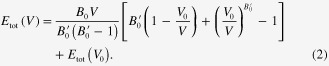
Here, 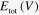 is the total energy of the system at volume *V*. 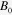 and 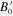 are the bulk modulus and its pressure derivative at the equilibrium volume 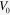, respectively. In the present study, the total energy was calculated for 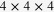 simple-cubic supercells with the lowest formation energies of the SQS models (see section 3) at nine different volumes for each concentration, after which the values were fitted.

The elastic moduli can be estimated by introducing the derivative of the energy as a function of lattice strain. For cubic systems, there are three independent elastic moduli, 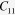, 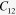, and 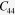, and it is possible to choose the strain in such a way that the volume of the cell is preserved [34]. To estimate 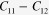, the volume-conserving orthorhombic strain tensor was introduced, which changes the total energy to 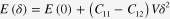. Furthermore, to estimate 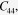 the volume-conserving monoclinic strain tensor was introduced, which changes the total energy to 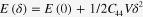. In the present study, *δ* = 0.01 was applied to obtain the elastic strain. Since the SQS model has low symmetry, strain was applied in 16 randomly selected directions, and the resulting values were averaged.

For isotropic cubic crystals, the bulk modulus is given by 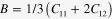. The shear modulus, *G*, and the Youngʼs modulus, *Y*, are related to the elastic constants by means of the following equations: 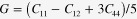 and 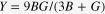.

### Hard x-ray photoelectron spectroscopy (HAXPES)

Five Ti–Mo base alloys with different chemical compositions, namely three Ti–Mo binary alloys (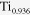 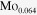 , 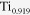 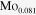, and 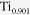 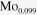 ) and two Ti–Mo–Fe ternary alloys 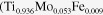 and 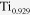 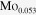 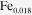 ) were selected for HAXPES measurements. Here, the atomic % values correspond to Ti-12, -15, and -18 mass % Mo, and Ti-10 mass % Mo-1 and -2 mass % Fe. The oxygen concentration of the present species are typically 0.27 at. % (the value is for the species 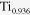 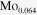) measured by inductively coupled plasma atomic emission spectroscopy (ICP-AES).

Ingots with a diameter of 70 mm and a weight of about 1 kg were prepared through cold-crucible levitation melting. Each ingot was hot forged and hot caliber rolled to 11.8 mm square bars. These bars were solution treated at 1073 K for 3.6 ks to obtain bcc single-phase microstructures. Plate samples (6 × 4 × 0.5 mm) were cut from the bars, and their surfaces were mechanically polished with diamond files.

The HAXPES measurements were performed at the BL15XU undulator beamline [35] at SPring-8. The photon energy was set to 5953.4 eV, which can lead to optimal energy resolution and photon flux of x-rays in combination with a Si(111) double-crystal monochromator and a Si(333) channel-cut crystal at the beamline. The x-rays were incident on the sample surface at a grazing angle of 2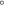. The kinetic energies of the photoelectrons were analyzed by a hemispherical electron analyzer (VG Scienta R4000). The E-vector of linearly polarized x-rays was set to be parallel to the direction of travel of photoelectrons entering the electron analyzer. The electron take-off angle as measured with respect to the surface was thus 88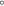, which maximized bulk sensitivity. The overall energy resolution was set to 240 meV, and the binding energy was referenced to the Fermi cut-off of an evaporated Au thin film. Since the Fermi cut-off energy was checked frequently against the Au thin film, the accuracy of the binding energy in all spectra was about ±10 meV.

## Results and discussion

The details of the SQS models of 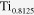 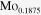, 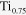 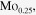 and 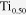 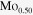 are given in tables 1, 2, and 3, respectively, for various calculation conditions. For each concentration, eight SQS models were constructed to perform first-principles calculations. Here, *f* is the number of clusters used in the evaluation of the correlation functions, and 
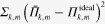
 is the sum of deviations from a perfectly random distribution of atoms. The value of 
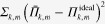
 might never become zero if the system size is large or the number of clusters increases. Furthermore, in general, 
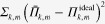
 increases with increasing the number of clusters *f*.

**Table 1.** Details of the SQS models for 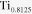 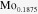.

	System size	*f*	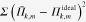	Formation energy, eV/atom
(I-a)	128	14	0.000 282	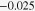
(I-b)	128	80	0.111 287	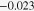
(I-c)	128	80	0.071 568	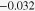
(I-d)	128	80	0.009 437	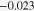
(I-e)	128	80	0.008 652	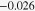
(I-f)	128	99	0.018 935	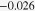
(I-g)	128	99	0.013 213	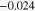
(I-h)	384	80	0.006 222	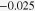

**Table 3.** Details of the SQS models for 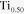 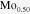 and B2 and B32 structures.

	System size	*f*	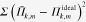	Formation energy, eV/atom
(III-a)	128	14	0.002 977	−0.087
(III-b)	128	80	0.077 374	−0.090
(III-c)	128	80	0.031 835	−0.095
(III-d)	128	80	0.024 150	−0.092
(III-e)	128	80	0.019 789	−0.089
(III-f)	128	99	0.026 532	−0.088
(III-g)	128	99	0.035 131	−0.093
(III-h)	384	80	0.008 698	−0.089
B2 structure	128	80	63.000	−0.051
B32 structure	128	80	40.000	−0.078

**Figure 1:**
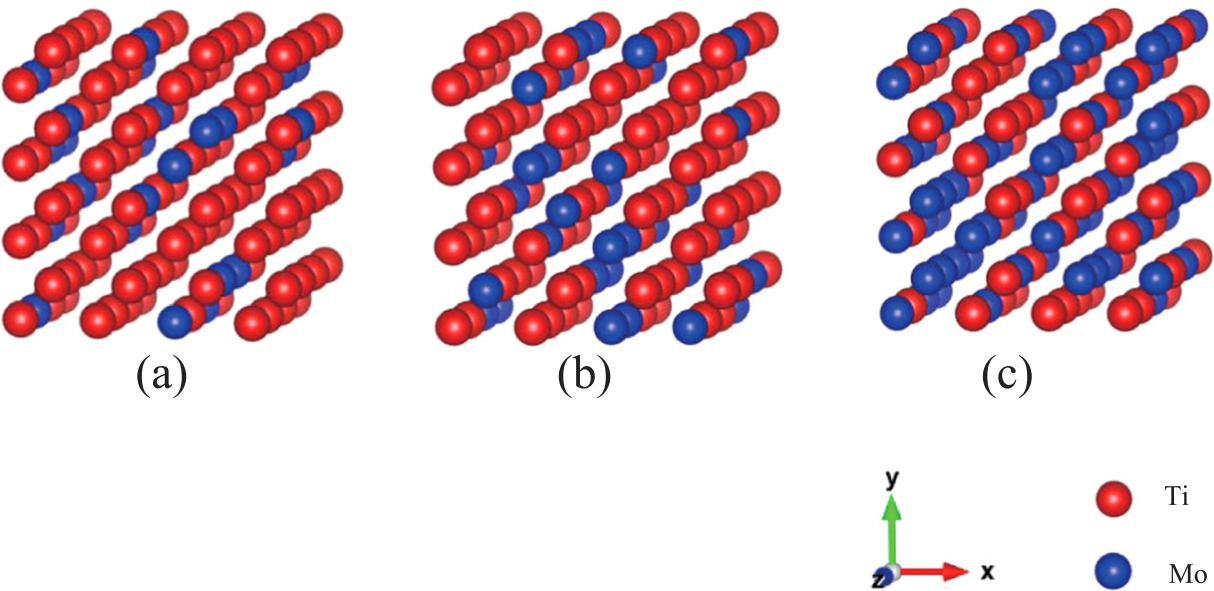
Examples of SQS models of 128-atom systems in the case of the lowest formation energies shown in tables 1–3. (a) 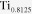

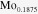
(I-c), (b) 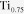

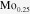
(II-b), and (c) 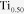

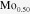
.

The structural and electronic properties of the alloys were analyzed using the SQS models. Figure 1 shows the SQS models for the 128-atom system. These are distributions at the lowest formation energies. Tables 1(c), 2(b), and 3(c) correspond to the distributions shown in figures 1(a), (b), and (c), respectively.

The correlation function is the index of the short-range ordering. In tables 1–3, we can see how the values, the sum of deviations from a perfectly random distribution of atoms, differ from the ideal solid solution for the case of the present SQS models as well as the ordered structures. It is clear that the deviation from the ideal solid solution for the SQS models are negligibly small compared with the case of the ordered structures. To the authorsʼ knowledge there exist no experimental evidence to support the existence of short-range ordered structures at room temperature. The short-range ordered structure usually produce diffuse scattering in electron diffraction patterns. But in Ti–Mo alloys, diffuse scattering observed is attributed to athermal Omega phase and not to the short-range order (SRO) [5].

**Figure 2:**
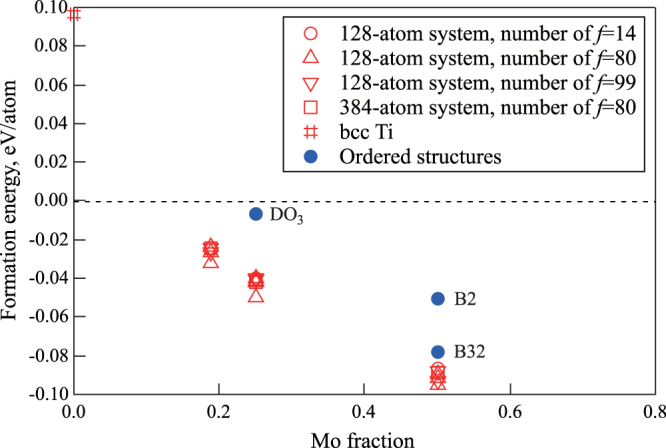
Mo concentration dependence of the formation energy of bcc alloys. Open and closed symbols denote the results for SQS and ordered structure models (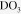 for 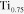 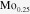, and B2 and B32 for 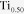 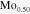), respectively.

Figure 2 shows the Mo concentration dependence of the formation energies per atom; these are listed in tables 1–3. The open symbols denote the results obtained using the SQS models; the closed circles denote the results of the ordered structure models, 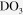 , B2, and B32.

Although the calculation conditions for constructing the SQS models are different, the differences in terms of formation energy are rather small. The differences between the highest and lowest formation energies are 0.008, 0.010, and 0.008 eV per atom in the case of 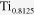 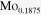, 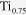 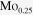, and 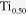 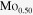, respectively. The differences in terms of system size are also negligible (figure 2). By comparing the formation energies of the SQS models with those of the ordered structure models, it was found that the disordered solid solutions in the SQS models are much more stable than those in the ordered structure models. The difference between the average values of the formation energies of the SQS models and the ordered structure models is 0.035 and 0.012 eV per atom for 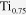 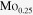 and 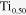 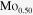, respectively. Therefore, Ti–Mo alloys are expected to form solid solution systems at room temperature with the mixture of the almost same lower energy configurations. Since the alloys are stable in the region with negative formation energy, bcc 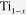 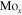 alloys should be stable for *x* higher than about 10%, which was evaluated by extrapolating the formation energies obtained by the SQS models by fitting the quadratic function.

**Figure 3:**
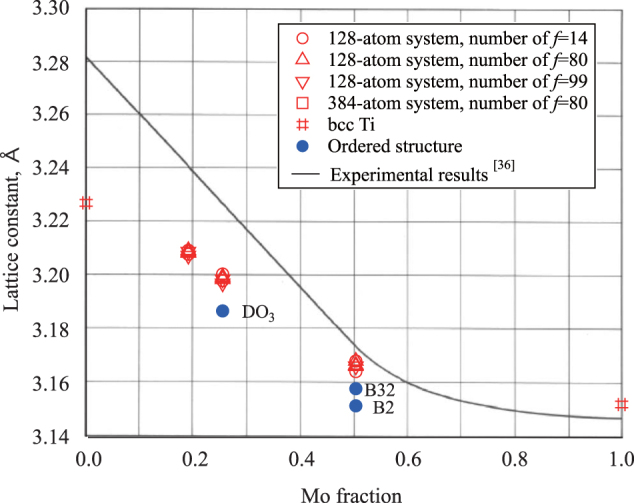
Mo concentration dependence of the lattice constant of the bcc alloys. Open and solid symbols denote the results for SQS and ordered structure models (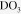 for 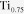 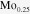, and B2 and B32 for 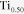 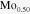), respectively.

Figure 3 shows the Mo concentration dependence of the lattice constants. Open and closed symbols stand for SQS models and ordered structure models, respectively. For comparison, the solid line represents the experimental results [38]. It is clear that the calculated lattice constant decreases monotonically with Mo concentration. For 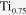 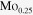 and 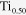 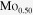, the lattice constants of the SQS models are larger than those of the ordered structure models and exhibit good agreement with the experimental values. The difference between the present calculations and the experimental values is about 0.7%, 0.9%, and only 0.2% for 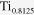 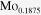, 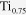 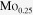, and 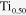 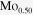, respectively. Furthermore, the calculation results do not obey Vegardʼs law, which is consistent with the experimental results. The calculated lattice constant of bcc Mo is larger than the experimentally determined value by about 0.2%, which is a general characteristic of GGA approximation. In contrast, the lattice constant of bcc Ti is smaller than the experimentally determined value by about 1.6%, and this is consistent with existing theory [9].

For all SQS models, there are noncubic distortions in the optimized structures, but these are usually negligibly small (about 0.08% in maximum). Since the formation energy differences among the SQS models for a particular *x* are small, as discussed above, the compounds are expected to show random distribution of Ti and Mo atoms at room temperature, which would lead to a perfect cubic lattice.

Table 4 shows the predicted elastic properties obtained using the SQS models. All the elastic properties increase with increasing Mo concentration, resulting in a stable bcc phase. The bcc phase is expected to become mechanically unstable and the hcp phase is expected to become stable at lower concentrations of Mo. It is important to predict the fundamental properties of materials such as the elastic properties. As an example of the application, in the case of Ti alloys, the properties are required to be controlled when biocompatible materials such as artificial bones are designed.

**Table 4.** Elastic modulus obtained for SQS models in units of GPa.

	Composition	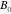	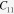	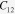	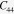	*G*	*Y*
(I-c)	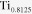 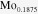	125.8	160.8	108.3	12.4	18.0	51.4
(II-b)	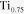 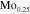	136.7	186.6	111.8	46.0	42.6	115.7
(III-c)	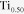 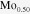	174.7	295.5	114.4	58.4	71.3	188.2

**Figure 4:**
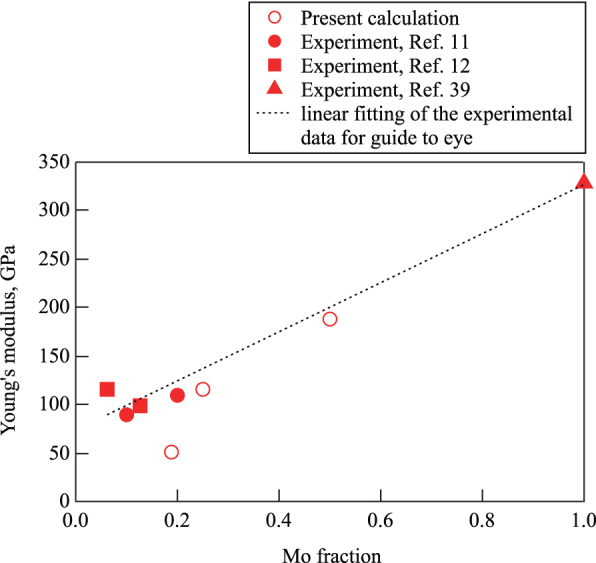
Mo concentration dependence of the Youngʼs modulus of the bcc alloys. Open symbols denote the calculated values and solid symbols denote the experimental values. The linear fitting of the experimental data is a guide to eyes.

Since it is expected that the elastic properties of *β* Ti–Mo alloys increase monotonically with Mo concentration [36, 37], figure 4 shows the relationship between the calculated Youngʼs moduli and Mo concentration with existing experimental data for comparison. In the figure, the dotted line is a linear fitting of the experimental data as a guide to the eye.

In all the cases, it is expected that the experimental Youngʼs moduli are larger than the theoretical estimation. The origin of the discrepancy is that the theoretical studies were restricted to bulk single crystals, while the real samples are polycrystalline [11]. In general, this tendency is the same as the existing theoretical works. The predictions by Raabe *et al* were lower than the experimental values for all the cases (see figure 6(b) in [11]). The predictions by Wang *et al* were 43.9 GPa and 78 GPa for 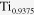 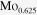 and 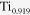 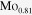, respectively (see table 2 in [12]). As for the predictions by Ikehata *et al*, even in the case of 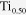 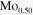, it was only about 50 GPa (see figure 2(d) in [9]). This is because their predictions were only for the ordered structures and different from the actual cases.

**Figure 5:**
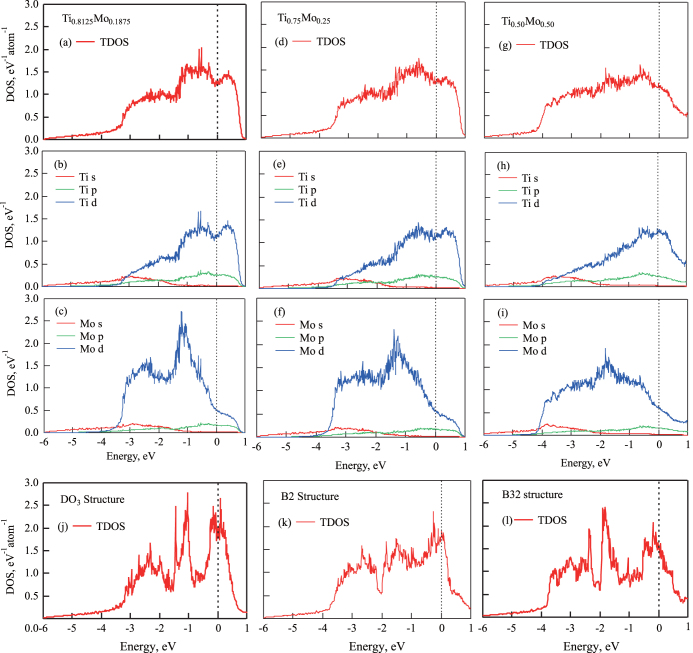
TDOS and site-projected PDOS for (a)–(c) 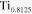 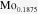, (d)–(f) 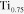 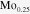, and (g)–(i) 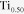 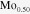 with the lowest formation energy in tables 1–3. For comparison, (j), (k), and (l) denote the TDOS for 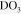, B2, and B32 structures, respectively. The Fermi level is at 0 eV.

Next, we studied the variation in electronic structure of the compounds with respect to the distribution and concentration of atoms. Figure 5 shows the total and site-projected partial density of states (PDOS) along with the angular momentum decomposition at Ti and Mo sites for (a)–(c) 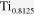 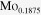, (d)–(f) 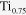 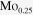, and (g)–(i) 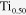 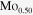, respectively. Panels (a), (d), and (g) show the total DOS (TDOS); (b), (e), and (h) show the PDOS at Ti sites; and (c), (f), and (i) show the PDOS at Mo sites, respectively. Again, these are the distributions with the lowest formation energies. For comparison, (j), (k), and (l) show the TDOS for 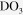, B2, and B32 ordered structures, respectively. The Fermi level is at 0 eV.

For all nonstoichiometric concentrations, the main contributors to the TDOS are d electrons of both Ti and Mo. The outlines of TDOS are clearly the same for all SQS models of the same concentration. In the case of low Mo concentration (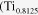 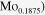), there is a small peak around 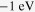, which comes from Mo d electrons. The peak decreases with increasing Mo concentration, and the outlines of TDOS and PDOS become flat. Furthermore, the shapes of TDOS and PDOS are clearly different from those of the ordered structure models (figure 5).

**Figure 6:**
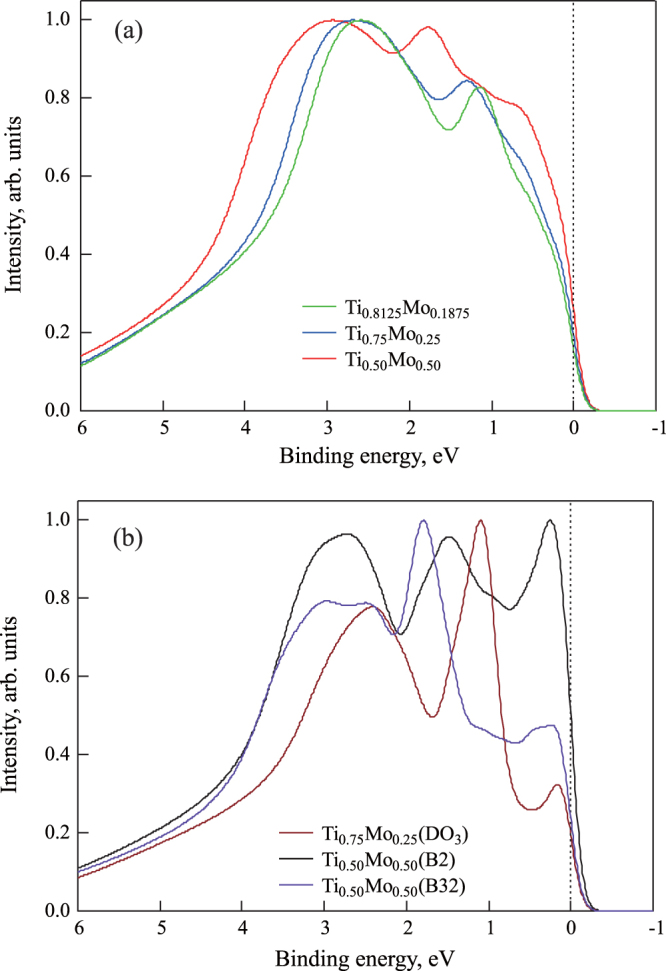
Calculated valence band HAXPES spectra for 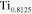 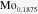, 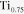 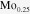, and 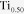 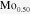. The Fermi level is at 0 eV.

To compare the electronic structures obtained with the SQS model and the experimental valence band spectra obtained by HAXPES, the calculated PDOSs were multiplied by the relative photoionization cross-sections. Here, the photoionization cross-sections for x-ray energy of 6 keV are the following (unit: 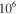 barn): Ti 4 s, 31.74; Ti 3d, 0.37760; Mo 5 s, 47.8176; Mo 4d, 17.3870 [39]. The Lorentzian function, Fermi–Dirac function at 300 K, and Gaussian function were introduced to consider the experimental resolution and lifetime extension. Figure 6 shows the calculated valence-band HAXPES spectra for (a) 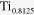 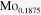, 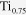 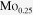, and 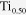 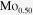 with the SQS approach, and (b)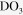, B2, and B32 ordered structure approach for comparison. The Fermi level is at 0 eV. The spectral intensities were normalized by the highest intensity in this binding energy region.

Figure 6(a) shows that there are two peaks within the binding energy region. The main peak (at around 3 eV of the binding energy) is originally from the 4s electrons of Ti. The other small peak (at around 1 eV of the binding energy in the case of lower Mo concentration) is originally from the 4d electrons of Mo. Both peaks shift to a higher binding energy with Mo concentration. Figure 6(b) shows that there are three sharp peaks within the binding region. The three peaks are dominated by the 4d electrons of Mo.

Before comparing the calculated and the experimental valence HAXPES data, we confirmed the configuration dependence of the calculated ones. That is, by comparing the results with the case of the SQS model for 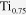 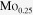 and 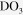 structure, and the case of the SQS model for 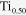 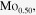 B2 and B32 structures, it is clear that the atomic distribution strongly affects the electronic structures and valence band spectra.

**Figure 7:**
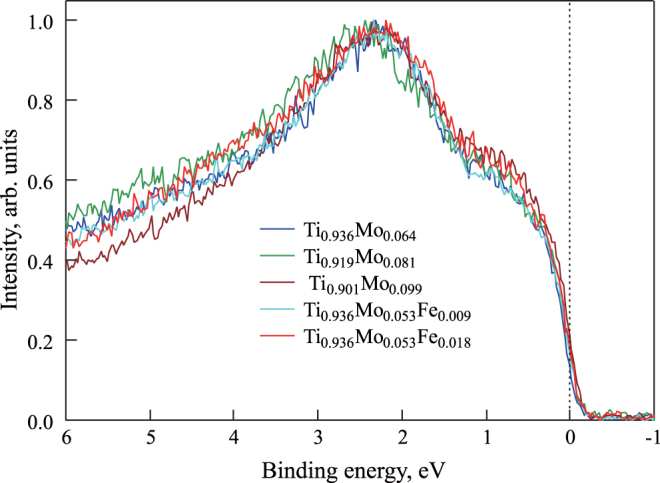
Experimental valence band HAXPES spectra for 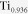 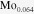, 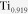 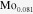, 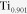 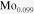, 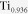 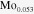 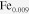, and 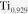 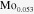 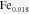 (which correspond to Ti-12 mass % Mo, Ti-15 mass % Mo, Ti-18 mass % Mo, Ti-10 mass % Mo-1 mass % Fe, and Ti-10 mass % Mo-2 mass % Fe, respectively). The Fermi level is at 0 eV.

Figure 7 shows the experimental valence band spectra for three Ti–Mo binary alloys (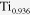 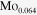, 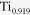 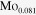, and 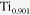 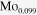), and for two Ti–Mo–Fe ternary alloys (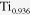 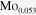 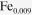 and 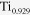 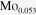 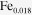). Again, the spectral intensities are normalized by the highest intensity in this binding energy region. It is noted that the effect of oxygen becomes clear in the higher energy regions such as the core region. However the effect is not so clear at the Fermi level.

Figure 7 shows that the intensity reaches a maximum at around 2.5 eV of the binding energy and increases with increasing Mo concentration in the vicinity of the Fermi level. Since the Ti–Mo alloys are solid solution of bcc lattice, it is expected that the shape of the spectra obtained changes continuously within the present Mo concentration range. Therefore, we compare between the calculated and the observed spectra in the different Mo concentrations (the difference is about 8 at.%). Comparing figures 6(a) and 7, we can see the similarity between the calculated and experimentally acquired HAXPES spectra. That is, looking at the calculated results for the compound with low Mo concentration (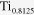 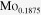), the results are comparable to those obtained experimentally: we can see a similarity in peak shape, although the peak positions are slightly different. The reason for this discrepancy might be that the calculation conditions for estimating the valence band spectra are different from the experimental conditions. Nevertheless, the calculated HAXPES spectra when using the SQS approach are in qualitative agreement with the experimental results, while the shapes of the calculated HAXPES spectra for the ordered structures are quite different from the shapes of the experimentally acquired spectra.

These results suggest that the randomness of atom distribution in bcc Ti–Mo alloys is important for simulating the experimental electronic and structural properties obtained by first-principles calculations. To study the solid solution system more accurately, it is necessary to consider the effect of SRO of atoms which results in the miscibility gap in the bcc lattice [40, 41], as quantitatively as possible. Therefore, in the end, we would like to note the limitation of the SQS models and the difference between the calculated model and the experimental observations. To the authors’ knowledge, no theoretical works exist which could reproduce the miscibility gap quantitatively in the bcc Ti–Mo alloys. In the SQS models, the atomic distribution were defined by considering only the geometrical conditions, that is, the atomic distributions were defined so that the deviation of the correlation functions from the ideal solid solution systems were negligibly small. This means that the model did not incorporate the effect of the SRO in the alloys. Since it is necessary to consider the effect of SRO when we treat the miscibility gap, the present models cannot reproduce it in the bcc Ti–Mo alloys. Thus, the calculated plots exhibit a downward convex in figure 2, which suggests a mixing tendency in the bcc lattice. So far, no information of the detailed atomic structure of SRO has been reported. If we have the detailed structure of SRO, we can construct a model to calculate the electronic structure.

## Summary

The structural and electronic properties of bcc Ti–Mo alloys were studied by first-principles calculations. In order to emulate the solid solution state of the alloys, the SQS approach was introduced in the first-principles calculations.

SQS models showed a much lower formation energy than ordered structure models. Lattice parameters calculated from the SQS models were closer to the experimental ones than those calculated from the ordered structure models. SQS models also reproduced the experimentally observed deviation from Vegardʼs rule.

The valence band photoelectron spectra simulated from the density of states obtained for random solid solution using the SQS models showed better agreement with the experimental data than those simulated from the density of states for ordered structure models.

These results indicate that the SQS model is effective for predicting the various properties of solid solution alloys by means of first-principles calculations.
